# Research progress on transcranial direct current stimulation for improving muscle strength: a narrative review

**DOI:** 10.3389/fneur.2025.1709933

**Published:** 2026-01-02

**Authors:** Yiguang Zhang, Yi Zhang, Yu Song, Huaduo Wu, Xin Gao, Yumo Dong, Ning Jiang

**Affiliations:** Tianjin Key Laboratory of Exercise Physiology and Sports Medicine, The Key Laboratory of Physical Health Integration and Health Promotion in Tianjin, Institute of Sport, Exercise & Health, Tianjin University of Sport, Tianjin, China

**Keywords:** transcranial direct current stimulation, maximum muscle strength, explosive power, muscle endurance, neuromuscular, motor unit recruitment

## Abstract

Muscle strength plays a fundamental role in enhancing sports performance, preventing sports injuries, and improving overall quality of life. In recent years, transcranial direct current stimulation (tDCS) has garnered significant attention in the field of motor performance enhancement. As a non-invasive brain stimulation technique, tDCS applies weak, constant direct current through electrode plates placed on the scalp to modulate neural excitability in specific areas of the cerebral cortex. However, the effects of tDCS on improving muscle strength remain inconsistent, and its exact mechanisms are still unclear. Further research is warranted to clarify its efficacy. This review summarizes research on the influence of tDCS interventions on muscle strength, focusing on maximum muscle strength, explosive power, and muscle endurance. It aims to analyze the outcomes of tDCS interventions across various sports tasks, and to discuss potential mechanisms through which tDCS may affect muscle strength. The collected evidence suggests that tDCS has the potential to influence muscle strength; however, considerable variations in its effects on athletes’ specific skills may be related to individual differences and varying stimulation protocols. This review consolidates existing evidence and offers relevant suggestions for future research, providing a theoretical foundation for the application of tDCS to improving muscle strength.

## 1 Introduction

Muscle strength refers to the ability of skeletal muscles to overcome internal and external resistance during activity. Based on the mechanical characteristics and temporal properties of muscular activity, strength can be categorized into three types: absolute strength (maximum muscle strength), defined as the peak force generated by muscles during a single contraction at a specific angle; speed strength (explosive power), which denotes the ability to produce maximum force impulse in a brief period; and strength endurance (muscle endurance), characterized by the capacity to sustain repeated or continuous muscle actions against a load. As an essential component of physical fitness, muscle strength plays a crucial role in enhancing sports performance, preventing sports injuries, and improving overall quality of life. With the increasing intensity of international sports competitions, the integration of science and technology has become increasingly important for boosting athletic performance, unlocking personal potential, and even challenging human limits. Transcranial direct current stimulation (tDCS) is a rapidly advancing neuromodulation technology that has garnered considerable interest in recent years, particularly in the field of sports performance enhancement.

tDCS is a non-invasive technique used to regulate neuronal activity in the cerebral cortex. It stimulates specific local cortical areas by delivering a constant, low-intensity current (typically 1 or 2 mA) through electrodes placed on the scalp, thereby modulating the neural excitability of targeted regions (1). Due to its low cost, high safety profile, ease of use, and reported neuromodulatory effects, tDCS has been widely utilized in neural rehabilitation (2) and cognitive science (3). In clinical applications, tDCS is considered promising. It has begun to be applied in neurological rehabilitation, particularly in facilitating motor recovery for stroke patients, by stimulating the motor cortex of the brain to enhance their motor functions (4, 5). In the treatment of mental and psychological illnesses, tDCS has significantly advanced research on depression and other conditions, providing new supportive methods for their treatment (6). In recent years, the application of tDCS has gradually shifted from rehabilitation to sports science. An intervention combining tDCS with physical training, conducted by the United States Ski and Snowboard Association with seven elite ski athletes, reported significant improvements in jumping ability and coordination in the anodal tDCS (a-tDCS) group compared to the sham tDCS (s-tDCS) group (7). Subsequent studies have suggested that tDCS may enhance neuromuscular connectivity, potentially increasing neuromuscular excitability and intermuscular coordination (8). However, not all studies support the efficacy of tDCS in enhancing muscle strength. The study conducted by Alix-Fages et al. (9) found that while tDCS can increase the number of repetitions in the bench press, it does not significantly improve maximal strength (one-repetition maximum, 1RM) or explosive power (force × velocity). Furthermore, Flood et al. (10) discovered that a-tDCS does not enhance the exercise duration of fitness enthusiasts. The commonly proposed mechanism underlying a-tDCS effects involves the depolarization of neuronal membrane potentials, which is believed to increase cortical excitability and facilitate neuronal activation (11). This heightened excitability may optimize motor unit recruitment and modulate synaptic plasticity (11), potentially improving intermuscular coordination (12) and the efficiency of force transmission, thereby contributing to enhanced athletic performance (13). Furthermore, a-tDCS has been suggested to modulate perceived fatigue (14), thereby influencing related neural pathways and neuromuscular control networks, which may have an impact on muscle strength (15). The failure of tDCS to enhance the subjects’ strength may be attributed to their anatomical structure and baseline neurophysiological characteristics (16, 17). Presently, the effects of tDCS on strength improvement remain inconsistent, and its precise mechanisms are not fully understood. Further research is necessary to clarify these effects. The aim of this study is to review the existing literature on how tDCS influences muscle strength, analyze advancements in studies regarding maximum muscle strength, explosive power, and muscle endurance, examine the proposed mechanisms and intervention strategies of tDCS on muscle strength, and provide both a theoretical foundation for utilizing tDCS to enhance muscle strength in subjects.

## 2 Overview of tDCS

In the early 19th century, the discovery of electrical nerve signal transmission led to the hypothesis that stimulating the brain with current could potentially enhance both motor and cognitive abilities. Research into the initial large-scale clinical application of transcranial electrical stimulation therapy for the treatment of severe mental illnesses commenced in the 1870s (18). However, it was not until the beginning of this century that tDCS technology gained broader recognition and acceptance (19), leading to its application in research and treatment of mental illnesses (20). Today, tDCS is widely used in sports training and competitions. It can be divided into a-tDCS, cathodal tDCS (c-tDCS) and s-tDCS, which serves to control for the “placebo” effect (19). Specifically, tDCS is a non-invasive technique that is generally regarded as safe and has shown potential for modulating brain function (21). By applying a low-intensity current through scalp electrodes, tDCS is hypothesized to modulate neural activity in targeted brain regions. This proposed modulation is then investigated for its possible effects on motor performance (22).

### 2.1 The mechanism of action of tDCS

**Figure 1:**
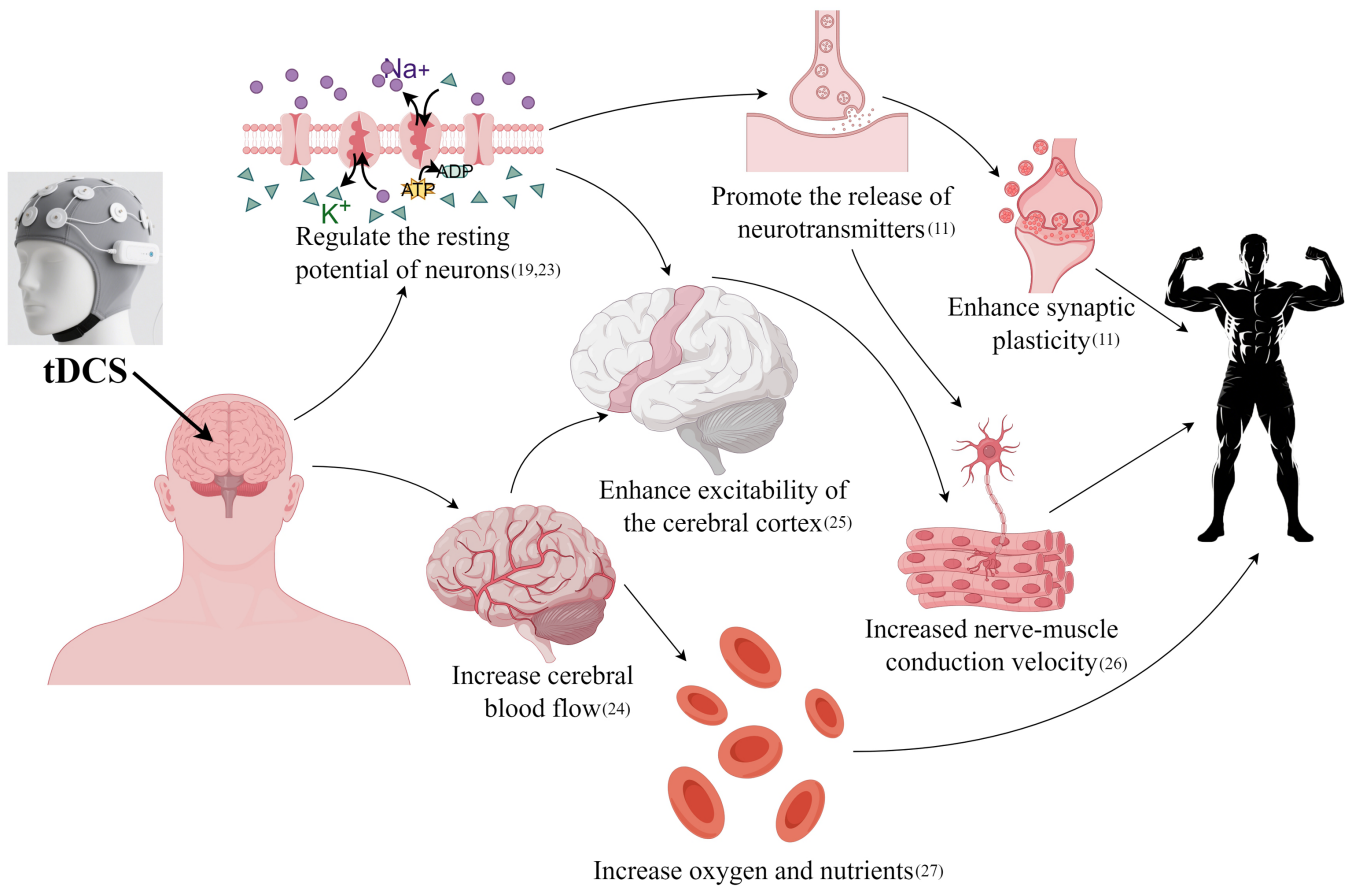
Potential mechanisms of tDCS in improving muscle strength. Previous studies have shown that tDCS may modulate neuronal resting membrane potential (19, 25) and increase cerebral blood flow (108), which could enhance cortical excitability (23). Alterations in the resting membrane potential are thought to promote neurotransmitter release and improve synaptic plasticity (11), thereby potentially increasing neuromuscular conduction velocity (135). The increase in cerebral blood flow may improve the delivery of oxygen and nutrients (32). These effects may collectively contribute to the enhancement of muscle strength.

As a non-invasive brain stimulation technique, tDCS is hypothesized to modulate muscle strength through the multi-level coordinated regulation of the central nervous system. We have traditionally posited that a-tDCS may increase cortical excitability by depolarizing neuronal membrane potentials, whereas c-tDCS might reduce excitability by inducing hyperpolarization. However, with advancing research, accumulating evidence indicates that the actual neurophysiological effects of tDCS are substantially more complex and subject to modulation by multiple factors, including current direction relative to neuronal orientation, individual anatomical differences, and baseline neural states. While the precise neurophysiological mechanisms through which tDCS may affect motor performance remain incompletely understood, several prevailing theories have been proposed. Figure 1 illustrates the mechanisms involved.

Numerous scholars have investigated the effects of tDCS on neuronal transmembrane potential. Nitsche et al. (19) employed motor evoked potential (MEP) amplitudes elicited by transcranial magnetic stimulation to evaluate tDCS aftereffects. Their findings indicated that a-tDCS increased MEP amplitudes by approximately 20%, while c-tDCS reduced them by a similar magnitude during the stimulation period. A subsequent pharmacological investigation by the same group (23) monitored MEP amplitudes following tDCS administration of carbamazepine (a voltage-gated sodium channel blocker), flunarizine (a voltage-gated calcium channel blocker), and dextromethorphan (an N-methyl-D-aspartic acid (NMDA) receptor antagonist). For short-term stimulation effects, their results suggested that neuronal aftereffects were primarily mediated by membrane polarization. Under placebo conditions, a-tDCS appeared to enhance cortical excitability while c-tDCS diminished it. Notably, carbamazepine abolished the excitability-enhancing effect of a-tDCS, and flunarizine substantially attenuated it, though neither channel blocker appeared to affect the inhibitory aftereffects of c-tDCS. For long-term stimulation aftereffects, different mechanisms appeared to be involved. Both a-tDCS and c-tDCS produced what appeared to be long-lasting excitability enhancement and suppression, respectively, and these enduring aftereffects were prevented by dextromethorphan, suggesting possible NMDA receptor dependency. This pharmacological profile was supported by Liebetanz et al. (24), who similarly proposed that tDCS induces polarity-specific shifts in neuronal membrane potential beneath the electrodes: a-tDCS may cause depolarization of the resting membrane potential, while c-tDCS might induce hyperpolarization. Notably, the neuromodulatory effects of the electric field may exhibit directional dependency (25). Modeling work by Manola et al. (26) suggested that when tDCS is applied over the motor cortex crown, anodal stimulation could preferentially activate pyramidal neurons with axons perpendicular to the cortical surface. Similarly, within the anterior wall and lip of the central sulcus, a-tDCS might preferentially activate neurons whose dendrites are oriented toward the electrode surface. Complementing this, Rahman et al. (27) used magnetic resonance imaging-derived finite element models and demonstrated that the tangential electric field (parallel to the soma-dendritic axis) tends to dominate in the cortex, with an intensity reportedly 4–12 times greater than the radial field (perpendicular to the soma-dendritic axis). This directional specificity might help explain observations that c-tDCS applied to the cerebellar cortex can paradoxically depolarize certain neuronal populations due to specific axonal orientations and potentially enhance motor learning efficiency (28). The mechanisms underlying potential long-term changes are thought to involve synaptic plasticity. Animal studies have reported that a-tDCS can enhance the amplitude of long-term potentiation (LTP) in mice (29). Furthermore, long-term tDCS has been shown to promote dendritic spine regeneration and synaptic stability, suggesting a potential mechanism for lasting functional adaptations within motor cortical circuits (29). Consistent with this, Kronberg et al. found that tDCS facilitates LTP, a phenomenon consistent with Hebbian plasticity principles (30). A leading hypothesis proposes that tDCS may modulate regional cortical excitability by influencing neuronal resting membrane potential. This modulated excitability could potentially enhance conduction efficiency along the corticospinal tract. For the observed long-term changes, it is theorized that by increasing neuronal excitability, a-tDCS might facilitate presynaptic glutamate release, thereby promoting activation of postsynaptic NMDA receptors. This sequence is believed to trigger calcium (Ca^2+^) influx, which could then induce LTP.

High-intensity strength activities require the brain to sustain an adequate energy supply, and the relationship between tDCS and cerebral blood flow has attracted research interest. Han et al. (31) used functional near-infrared spectroscopy (fNIRS) to monitor tDCS effects on oxyhemoglobin levels in rats and reported nearly linear increases during stimulation and decreases afterward. Xia et al. (32) first documented that tDCS can transiently alter blood–brain barrier permeability in rats, significantly increasing the effective diffusion coefficient of solutes in brain tissue, with a more pronounced effect on large molecules than on small ones. Antal et al. (33) examined how short-term tDCS over the primary motor cortex (M1) influenced brain activity during rest and finger-tapping tasks, finding that tDCS did not alter resting-state blood oxygen levels, but a-tDCS modulated them during the task. Giorli et al. (34) investigated the effect of tDCS on cerebrovascular vasomotor reactivity (VMR)—the ability of cerebral arterioles to change their diameter in response to stimuli—in healthy subjects. Their results indicated that a-tDCS reduced VMR and increased mean blood flow velocity at rest, while c-tDCS produced opposite effects, with sham stimulation showing no significant impact. Additionally, Shinde et al. (35) observed that a-tDCS applied to the M1 increased cerebral blood flow in targeted subcortical areas, and higher stimulation intensities induced more pronounced changes. Taken together, these findings suggest that tDCS may influence hemodynamics in both cortical and subcortical regions by modulating local cerebral blood flow, and such changes appear correlated with alterations in cortical excitability. The brain operates as a complex network system, with various regions working collaboratively to regulate multiple physiological functions including movement, cognition, and emotion. One functional magnetic resonance imaging study reported that applying a-tDCS over M1 or the dorsolateral prefrontal cortex (DLPFC) was associated with enhanced functional connectivity between related brain regions and improved cognitive performance in subjects (36). Another stimulation study targeting the cerebellar Crus II region found that a-tDCS significantly increased long-range functional connectivity between the right cerebellar Crus II and the right inferior frontal gyrus (37). In research involving competitive swimmers who received a-tDCS over the DLPFC, stimulation was observed to reduce local functional connectivity within the stimulated left hemisphere. The authors hypothesized that this reduction in local connectivity might reflect a shift towards stronger functional coupling with distal cortical nodes, which could in theory promote the integration of large-scale cognitive networks. Notably, a-tDCS was linked to reduced lap times during the 25–50 meter segment of a 100-meter swim. The observed decrease in local connectivity may correlate with this improvement in athletic performance (38). Milton et al. (39) demonstrated that brain activity during pre-shot routines in expert golfers differs substantially from that in novices, suggesting that extensive long-term practice leads to a more efficient neural organization focused on task-relevant networks in skilled athletes. Therefore, tDCS might aid athletes in maintaining cognitive control and skill execution more effectively, possibly by reducing redundant or inefficient local co-activation. This points to a potential mechanism by which tDCS could modulate functional brain network connectivity, thereby supporting enhanced motor performance.

In contrast to the proposed mechanism of a-tDCS, c-tDCS is not thought to enhance muscle strength by directly amplifying the motor command to the target muscle. Instead, its effects are primarily attributed to indirect modulation of nervous system function, which is highly dependent on the stimulation parameters. Currently, the majority of studies have used c-tDCS in the treatment of neurological and psychiatric disorders (40–42). However, some studies have found that c-tDCS has the potential to improve muscle function strength (43). When the brain signals for movement, it requires the body’s agonistic muscles and antagonistic muscles to work together. When the agonistic muscles contract to produce action, the antagonistic muscles must relax accordingly (i.e., interactive inhibition) (44, 45). Studies have shown that c-tDCS may improve the cross-inhibition of healthy individuals (46), thereby selectively reducing the excitation level of antagonistic muscles (43). Complementary evidence from studies applying a-tDCS shows a reduction in cross-inhibition from wrist flexor to extensor (47), supporting the role of tDCS in modulating these inhibitory neural circuits. It is believed that c-tDCS may function by decreasing the excitability of the cerebral cortex and lowering the motor evoked potential in the antagonistic muscle. This suppression in resistance from the antagonistic muscle during exercise may aid in optimizing the neural drive pattern, resulting in increased agonistic muscle strength and improved exercise efficiency. The influence of c-tDCS on muscle strength may also occur through the modulation of activity between the brain’s hemispheres. In stroke rehabilitation, c-tDCS is frequently used to suppress the overexcitation of the unaffected hemisphere, thereby contributing to the rebalancing of interhemispheric activity inhibition (48–50). Its inhibition may lead to abnormal or overly active brain activity, and c-tDCS may improve the function of the M1 of the target muscle by suppressing inhibitory responses, thereby improving strength output. The effect of c-tDCS on muscle strength follows a nonlinear relationship between stimulation intensity and intervention outcome. Studies have reported that 1 mA and 1.5 mA c-tDCS can reduce muscle strength, whereas 2 mA stimulation has been observed to increase it (51). Further investigating the interaction between intensity and duration, one study examined sham, 1 mA, 2 mA, and 3 mA stimulation combined with durations of 15, 20, and 30 min. Results indicated that LTP was induced specifically in the 2 mA-20 min group, while the 1 mA-15 min, 1 mA-30 min, and 3 mA-20 min protocols all resulted in long-term depression (LTD) (52). This reversal of effect may be associated with dynamic fluctuations in neuronal Ca^2+^ levels (53). It is proposed that low-intensity stimulation may induce mild Ca^2+^ influx, triggering LTD, whereas the 2 mA stimulus might cause a larger Ca^2+^ influx that surpasses the threshold required for LTP induction (52). A study using the Ca^2+^ channel blocker flunarizine found that the direction of tDCS-induced neuroplasticity is Ca^2+^-dependent (53). Furthermore, stimulation intensity appears to influence synaptic input characteristics. Within a certain range, weaker stimulation induces correspondingly weak synaptic input, with higher intensities leading to stronger and earlier Ca^2+^ pulses. When the stimulation intensity is too high, it induces strong synaptic input. In this case, because of the low sensitivity of the Ca^2+^ ion pulse to the field effect, the polarization of the cell body primarily influences the pulse timing, potentially explaining why very high currents fail to induce LTP (54). Additionally, a recent study exploring c-tDCS pretreatment prior to a-tDCS intervention found that this sequential protocol did not produce additive effects (55). Thus, the influence of c-tDCS on muscle strength exhibits a nonlinear relationship between stimulation intensity and intervention outcomes, which may arise from dynamic fluctuations in neuronal Ca^2+^ levels.

Beyond the mechanisms previously discussed, additional research has been conducted. One study found that the effects of a-tDCS do not vary linearly with stimulation duration. While 22 and 24 min of a-tDCS were observed to reduce GABAergic inhibition and increase glutamatergic excitation, stimulation exceeding 26 min paradoxically produced opposite effects, ultimately suppressing corticospinal excitability (56). The authors suggest this phenomenon may be related to the Bienenstock-Cooper-Munro rule. The aforementioned study by Mosayebi-Samani et al. (53) on c-tDCS stimulation intensity and duration reported similar non-linear patterns. Bienenstock et al. (57) found that the bidirectional induction threshold for synaptic plasticity (LTP/LTD threshold) is not fixed but dynamically adapts to synaptic activity levels. Based on these findings, we speculate that when interventions like tDCS cause sustained increases in cortical excitability, homeostatic mechanisms may be triggered. These mechanisms potentially enhance inhibitory synaptic transmission, inducing counter-adaptive changes that counteract the excitatory effects of tDCS and maintain network stability. Investigating the inconsistent effects of tDCS on learning, Lu et al. (58) utilized a homeostatic structural plasticity model. Their findings indicated that applying uniform tDCS, whether anodal or cathodal, during or after learning weakened the connection strength of motor engrams. This suggests that when tDCS-induced stimulation does not align with neural activity patterns generated during motor learning, it may interfere with or occlude the natural plasticity processes essential for learning, thereby blocking the intended effects of tDCS. In summary, the effects of tDCS interventions may also be influenced by homeostatic plasticity or occlusion effects. However, the precise mechanisms of tDCS action remain incompletely understood and warrant further investigation.

### 2.2 Stimulation parameters

The selection of stimulation parameters is a critical factor in tDCS research, as evidenced by significant variations in protocols across studies. These parameters directly influence both the putative neuromodulatory outcomes and the safety profile of the intervention. Existing studies reveal significant variations in the selection of key parameters, including current intensity, stimulation duration, and electrode size. Current intensity directly influences the stimulation effect, although excessively high current levels can increase the risk of adverse effects. Studies have shown that 2 mA stimulation may enhance motor cortex excitability by promoting synaptic plasticity and has a delayed effect of 60–90 min, though it may also cause scalp tingling. In contrast, 1 mA stimulation is generally better tolerated but has been found to be less effective than 2 mA in improving performance on certain cognitive tasks (59). Further research has investigated the feasibility of ultrahigh intensity stimulation at 4 mA, suggesting that it may potentially double synchronization in prefrontal nerves, though it could also raise the risk of skin erythema (60). The discussion of stimulation duration centers on the time dependence of neural plasticity. LTP can be induced with 20 min of stimulation, resulting in effects that persist for over 90 min; however, stimulation durations exceeding 30 min may lead to synaptic inhibition (61). Additionally, a threshold effect appears to exist between stimulation duration and current intensity. When the current is ≥ 2 mA, 20 min of stimulation saturates synaptic plasticity, whereas at 1 mA, up to 30 min could be required to elicit a comparable effect (62). Guidelines from the European Federation of Clinical Neurophysiology indicate that repeated short-term stimulation sessions tend to produce more favorable cumulative outcomes in rehabilitation settings (25). Electrode size also influences both stimulation efficacy and health risks. Although larger electrodes can significantly reduce injury risk, they lower current density and may diminish stimulation effectiveness. Some studies have shown that an electrode of 48 cm^2^ does not improve vertical jump height (63). Computer modeling has confirmed that a smaller 5 cm × 5 cm electrode can increase the electric field intensity in the target brain region (64), whereas a 35 cm^2^ electrode reduces current density within the effective area compared to a 25 cm^2^ electrode, which potentially decreases adverse reactions (65).

The effects of tDCS are influenced not solely by a single stimulus parameter but rather by the interactions among multiple parameters. When a 2 mA current is applied with a 35 cm^2^ large area electrode, the electric field distribution in the prefrontal cortex will expand compared to the parameters of 1 mA/25 cm^2^ (66), while using a 16 cm^2^ small area electrode with 30 min long-term stimulation might cause excessive activation of the cortical inhibitory network (67). However, these results also vary due to differences in anatomical structures like skull thickness and baseline neurophysiological conditions between individuals. These variations can result in weak or absent responses to tDCS in certain individuals, thereby diminishing the overall effectiveness of the intervention and complicating the observation of differences across diverse populations (17).

### 2.3 Stimulation area

The selection of target areas for tDCS is a crucial factor in determining its neural regulation specificity and functional effects. Different stimulation positions in various brain regions cause significant differences in physiological responses and behavioral outcomes. Currently, the M1, prefrontal cortex, and cerebellum are the most frequently targeted regions for tDCS. The M1, a well-established target, plays a vital role in generating nerve impulses that control human movement. Anodal stimulation applied to M1 has been shown to enhance the excitability of the corticospinal pathway, which may contribute to improvements in motor performance and learning (68). In contrast, the prefrontal cortex has been associated with cognition, emotion, pain, and behavior regulation. A comparative study of a-tDCS intervention in the bilateral DLPFC showed that left DLPFC stimulation with anode could enhance working memory, while right DLPFC cathode stimulation was more effective for emotion regulation (69). Numerous studies have demonstrated that a-tDCS stimulation of the DLPFC may improve motor performance (10, 70, 71). The cerebellum, regarded as an important center for movement regulation, has been shown to respond to anodal stimulation with improved motor coordination, though such stimulation does not appear to significantly influence the speed of simple motor responses (72). This may be related to the specific role of the cerebellum-thalamus-cortex loop in motion control, which adjusts motor output by comparing motor intention with actual execution, without directly affecting conduction velocity in the primary motor pathway (73, 74). Currently, some researchers are overcoming the limitations of single-target stimulation by exploring multi-target collaborative protocols. Synchronized stimulation of M1 and DLPFC has been shown to enhance the learning of complex motor tasks beyond what single-target methods can achieve. This improvement may be attributed to synergistic effects that enhance functional connectivity between the prefrontal and motor cortices (75). Additionally, high-density tDCS (HD-tDCS) can focus the electric field, reduce activation of non-target brain regions, and increase activation of the targeted areas (65). Future research should incorporate dynamic functional network analysis, such as near-infrared brain imaging, to develop multi-target sequential stimulation strategies that address the limitations of single-target regulation.

## 3 The effect of tDCS on muscle strength

The quality of strength directly determines the body’s ability to efficiently perform key actions such as acceleration, confrontation, and maintaining the stability of technical movements in most sports. Whether in sprinting, throwing, or weightlifting, which require extreme strength and speed; rowing and cycling, which emphasize continuous confrontation and endurance; or ball games and combat sports, which demand quick bursts of energy and overall strength, high-level strength serves as the biological foundation for athletes to excel in high-intensity competitions. Recently, tDCS has garnered interest as a potential technique for enhancing motor function through neurobiological modulation (76, 77). Studies suggest that tDCS may increase the excitability of the M1 and facilitate the acquisition of motor skills (78, 79). Additionally, it has been observed to reduce reliance on secondary motor areas and appears to lower the perceived effort during the performance of physical tasks (80). Research integrating tDCS with neuroimaging has provided further insights into its mechanisms. For instance, fNIRS data indicate that tDCS can improve neural transmission efficiency in the bilateral sensorimotor cortex (81) and may enhance muscle strength through increased corticospinal excitability (82). Collectively, these findings suggest a potential role for tDCS in supporting strength training and athletic performance.

### 3.1 The effect of tDCS on maximum muscle strength

Maximum muscle strength refers to the ability of the human neuromuscular system to overcome the maximum external resistance at a single moment through voluntary contraction under specific joint angles and movement modes. The level of muscle strength is highly related to motor unit recruitment. As a neuromodulation technique, a-tDCS technology has been proposed to increase the excitability of the cerebral cortex, which may in turn regulate neuron firing rates, accelerate the transmission of neural impulses to muscles, and improve motor unit recruitment, potentially leading to increases in muscle strength.

In studies investigating the effects of tDCS on maximum muscle strength, most studies have utilized 1RM or maximum voluntary contraction (MVC) of isolated movements as outcome measures (Table 1). Kamali et al. (83) administered 2 mA, 13 min tDCS sessions to 12 bodybuilders and observed improvements in the mean square root values of leg flexion and extension 1RM, along with a significant enhancement in quadriceps femoris performance post-intervention. In a study involving 20 female soccer players, Vargas et al. (84) applied 2 mA, 20 min a-tDCS and reported an increase in knee joint MVC compared to baseline measurements. Similar findings were reported by Ma et al. (85) in a group of professional rowers. Using a 2 mA, 20 min tDCS protocol, they noted a significant increase in peak torque of the left knee joint at 180°/s, which was accompanied by elevated neural activity in the right precentral gyrus. There are similar studies on the maximum muscle strength of the upper limbs. Hazime et al. (86) examined the influence of tDCS on shoulder internal/external rotation muscles in handball players and found that maximum voluntary isometric contraction (MVIC) was higher immediately and 30 min after the a-tDCS intervention compared to the sham tDCS group. Moreover, the muscles responsible for shoulder internal rotation continued to demonstrate greater strength 60 min post-stimulation. Some studies have also reported cross-activation effects following tDCS intervention. In a randomized, double-masked, crossover study involving 13 healthy adults, Frazer et al. (87) compared a-tDCS with s-tDCS, administered 1 week apart, in conjunction with right upper limb strength training. They observed that the maximum muscle strength of the right biceps was greater after a-tDCS than after s-tDCS, with a similar trend noted on the untrained left side. Additionally, corticospinal excitability and cross-activation were found to be elevated following a-tDCS relative to the sham condition. These findings raise the possibility that tDCS may help mitigate bilateral muscle strength imbalances and potentially lower the risk of sports injuries associated with such asymmetry. Relevant studies have shown that the dominant motor cortex may sustain its primary role in motor control by inhibiting the activity of the contralateral hemisphere (88). When applying a-tDCS to the M1 area, the dominant side may counteract some of the stimulation effects by inhibiting the input to the non-dominant cortex, while the non-dominant side will show more significant improvement due to weakened activity inhibition (89). This study suggests that the neuromuscular system on the non-dominant side is in an “underdeveloped” state due to infrequent use, and its synaptic plasticity and nerve remodeling potential are higher. Once inhibition is reduced, the effectiveness of strength training is improved. However, not all studies support the efficacy of tDCS in enhancing maximum muscle strength. For example, Alix-Fages et al. (9) found that a-tDCS increased repetitions at 75% 1RM in bench press but had no significant effect on 1RM or explosive power (strength × speed). Flood et al. (90) observed that a-tDCS did not improve MVC in the isokinetic muscle strength test of 12 healthy adults. Similarly, Maeda et al. (91) reported no significant differences between the a-tDCS and s-tDCS groups after 3 weeks of a-tDCS combined with lower limb strength training.

**Table 1:** The characteristics of relevant literature on the effect of tDCS on maximum muscle strength.

Authors	Study design	Blinding (participant/assessor)	Control type	Sample size	Age (years)	Stimulation parameters				Test scheme	Reported effect size (primary outcome)
						Electrode position	S (cm^2^)	I (mA)	*t* (min)		
Kamali et al. (83)	Crossover RCT	Double-blind (Yes/Yes)	s-tDCS	12 M	18–40	A: M1 (Cz) & T3; C: right shoulder	35	2	13	Knee extension 1RM	1RM: 4.4% improvement (*p* = 0.002). ES: Not reported
Vargas et al. (84)	Crossover RCT	Double-blind (Yes/Yes)	s-tDCS	20F	16.1 ± 0.8	A: M1 (C3/C4); C: ipsilateral supraorbital region	35	2	20	MVIC	Dominant limb MVIC (a-tDCS vs. s-tDCS): During tDCS: Cohen’s d = 0.6 (95% CI: −0.1 to 1.2). 30 min post-tDCS: d = 0.9 (95% CI: 0.2 to 1.5). 60 min post-tDCS: d = 0.95 (95% CI: 0.3 to 1.6)
Ma et al. (85)	Pre-post Study	No Blinding (No/No)	No Control Group	12 M	16	A: M1; C: -	-	2	20	Knee/Shoulder isokinetic muscle strength	Left knee/shoulder isokinetic muscle strength: significantly increased (*p* < 0.01) ES: Not reported
Hazime et al. (86)	Crossover RCT	Double-blind (Yes/Yes)	s-tDCS	8F	19.65 ± 2.55	A: M1; C: ipsilateral supraorbital region	35	2	20	Shoulder external/internal rotation, MVIC	Dominant limb MVIC (a-tDCS vs. s-tDCS) During tDCS: ~10.2% improvement; 30 min post-tDCS: ~18.6% improvement; 60 min post-tDCS: ~19.3% improvement. ES: Not reported
Frazer et al. (87)	Crossover RCT	Double-blind (Yes/Yes)	s-tDCS	13	18–35	A: Ipsilateral M1; C: Contralateral supraorbital	A: 25; C: 35	2	20	Elbow flexion and extension	Untrained limb 1RM (a-tDCS + ST vs. s-tDCS + ST): 12% vs. 2% increase ES: Not reported
Alix-Fages et al. (9)	Crossover RCT	Double-blind (Yes/Yes)	s-tDCS/c-tDCS	14 M	22.8 ± 3.0	A: left DLPFC; C: right orbitofrontal cortex	7.6 × 7.6 cm	2	15	bench press	1RM: No significant changes (*p* ≥ 0.377). ES: Not reported
Flood et al. (90)	Crossover RCT	Single-blind (Yes/No)	s-tDCS	12 M	18–40	A: M1 (C3/C4), C: Cz, F3/F4, T7/T8, P3/P4	HD-tDCS	2	20	MVC	MVC: No significant changes ES: Not reported
Maeda et al. (91)	Parallel RCT	Triple-blind (Yes/Yes; Data Analyst: Yes)	s-tDCS	24 (12F, 12 M)	23.7 ± 1.3	A: M1; C: Ipsilateral upper arm	25	2	10	Knee extension/flexion peak torque	Peak Torque (a-tDCS + training vs. s-tDCS + training): No significant difference between groups ES: Not reported

S, surface area; I, current intensity; t, time; RCT, randomized controlled trial; M, Males; F, Females; A, anodal; C, cathode; M1, primary motor cortex; T3, C3, C4, Cz, F3, F4, T7, T8, P3, P4, The electrode positions in the International 10/20 electroencephalography system; DLPFC, dorsolateral prefrontal cortex; 1RM, 1 Repetition Maximum; MVIC, maximum voluntary isometric contraction; MVC, maximum voluntary contraction; ES, effect size.

The improvement of maximal muscle strength appears to be associated with the recruitment of motor units. Research suggests that a-tDCS may enhance muscle strength performance by modulating cortical excitability and potentially contributing to more synchronized recruitment of motor units. Discrepancies in findings across studies may be attributed to variations in stimulation parameters, testing methodologies, and participant populations. Further research is needed to examine how tDCS improves maximum muscle strength specifically. Future studies should focus on optimizing stimulation parameters and explore the underlying causes of variability in results.

### 3.2 The effect of tDCS on explosive power

Explosive power, defined as the ability of the human body to produce maximum force within a brief time frame, directly determines the power output of the sports system and is crucial in various sports (92). The neuromuscular system primarily coordinates the control of explosive power. Insufficient explosive power leads to a slower rate of force development and delays in the transmission of joint torque. These changes significantly impact athletes’ power output and movement stability during explosive actions (93), and they also affect the ability of ordinary individuals to control sports activities under sudden loads.

Currently, most investigations into the effects of tDCS on muscle explosive power employ various jumping and sprinting tests (94–97) (Table 2). Grosprêtre et al. (98) applied 2 mA, 20 min a-tDCS to 18 athletes to evaluate their vertical jump and standing long jump, while also recording surface electromyography. Their results indicated that, following a-tDCS intervention, vertical jump height, standing long jump distance, and root mean square values of the tibialis anterior and gastrocnemius muscles showed improvement, with values appearing higher than those in the s-tDCS group. Lattari et al. (99) used the reverse vertical jump test to assess 10 strength lifters and found their jump height, stagnation time, and peak power significantly increased after stimulation. Similarly, Codella et al. (100) applied 2 mA, 20 min a-tDCS to 17 healthy adults, also observing that the vertical jump height of the a-tDCS group was significantly higher than that of the s-tDCS group post-stimulation. Previous studies suggest that the influence of a-tDCS on explosive power may have a post-effect. Jiang (101) tested 21 healthy adults with 2 mA, 20 min a-tDCS and found that vertical jump height increased immediately after intervention, with effects lasting at least 60 min. Gao et al. (102) used a force platform to examine the height of repeated vertical jumps and biomechanical performance of lower limbs following a-tDCS. The total height of repeated vertical jumps in the a-tDCS group was significantly higher than pre-stimulation immediately and 30 min afterward, with peak pedal power, power rate, and vertical impulse also significantly increased. Active muscle work increased significantly only immediately post-stimulation. Similar methods have been applied to sprint testing. Chen et al. (103) found that in the a-tDCS group, the vertical jump height without load and average sprint time were significantly higher than in controls, assessed via loaded vertical jump and repeated sprints. Regarding cycling and sprinting, Sasada et al. (104) reported that after 23 athletes received simultaneous stimulation of M1 and DLPFC regions, the average power during 30 s of maximal effort riding and sprinting was higher in the a-tDCS group than in the s-tDCS group. Huang et al. (105) stimulated 9 healthy adults with 2 mA for 20 min, observing significant improvements in peak power and average power during cycling sprints, along with a notable reduction in reaction time.

**Table 2:** The characteristics of relevant literature on the effect of tDCS on explosive power.

Authors	Study design	Blinding (participant/assessor)	Control type	Sample size	Age (years)	Stimulation parameters				Test scheme	Reported effect size (primary outcome)
						Electrode position	S (cm^2^)	I (mA)	*t* (min)		
Grospretre et al. (98)	Crossover RCT	Double-blind (Yes/Yes)	s-tDCS	18 M	22.6 ± 5.7	A: left PFC; C: right PFC	35	2	20	CMJ, SJ SLJ	CMJ: η^2^ = 0.186 (*p* = 0.0038); SJ: η^2^ = 0.262 (*p* = 0.0001); SLJ: η^2^ = 0.376 (*p* < 0.0001). Percentage improvements: SJ + 5.3%, CMJ + 4.0%, SLJ + 9.3%.
Lattari et al. (99)	Crossover RCT	Double-blind (Yes/Yes)	s-tDCS	10	18–28	A: motor cortex bilaterally; C: orbitofrontal cortex	35	2	20	CMJ	CMJ height: Effect size d = 1.22; Flight time: d = 1.22; Peak power: d = 1.22 60 min post-tDCS: d = 0.95 (95% CI: 0.3 to 1.6)
Codella et al. (100)	Crossover RCT	Single-blind (Yes/No)	s-tDCS	17	30.9 ± 6.5	Halo Sport headphones: C1-C6	28 (per electrode)	2	20	VJ	Vertical jump: η^2^ = 0.41 (*p* = 0.004)
Jiang et al. (101)	Crossover RCT	Double-blind (Yes/Yes)	s-tDCS	21 M	21–25	A: M1, C: ipsilateral shoulder	35	2	20	CMJ,	CMJ height: increased 1.22 cm post anodal (*p* < 0.05). ES: Not reported
Gao et al. (102)	Parallel RCT	Double-blind (Yes/Yes)	s-tDCS	30 M	20–25	A: bilateral M1; C: ipsilateral shoulder	A: 25; C: 35	2	20	60s-RVJT	total jump height: significantly higher post anodal (*p* < 0.05). ES: Not reported
Chen et al. (103)	Crossover RCT	Single-blind (Yes/Not stated)	s-tDCS	13 M	20.3 ± 0.6	Halo Sport headphones: CZ, C5, C6	7.6 × 7.6 cm	2	15	CMJ test, weighted CMJ test and repeated Sprints	CMJ height: significantly increased (*p* = 0.04); sprint time: significantly decreased (*p* = 0.016); ES: Not reported
Sasada et al. (104)	Crossover RCT	Double-blind (Yes/Unclear)	s-tDCS	13	18–40	A: vertex; C: right forehead	35	2	15	30-s maximal-effort sprint cycling	a-tDCS showed no significant improvement vs. s-tDCS. ES: Not reported
Huang et al. (105)	Crossover RCT	Triple-blind (Yes/Yes)	s-tDCS	9 M	23.7 ± 1.3	Halo Sport headphones: CZ, C5, C6	24 (per electrode)	2	10	5*6 s sprint cycling	Mean power output increased under Halo condition (ES = 0.60).
Park et al. (110)	Crossover RCT	Single-blind (Yes/Unclear)	s-tDCS	13F	21.92 ± 2.81	A: Cz; C: C5/C6	28 (per electrode)	2	20	VJ and spiking speed	Spike ball speed increased by 7.5% (η_p^2^ = 0.383, *p* < 0.001); No significant effects on VJ
Mesquita et al. (111)	Crossover RCT	Single-blind (Yes/Unclear)	s-tDCS	19	19 ± 3	A: C3 and C4; C: ipsilateral shoulders	A: 35; C: 25	1.5	15	CMJ and frequency speed of Kick test	FSKT total kicks: significantly lower in a-tDCS vs. s-tDCS (η_p^2^ = 0.394, large, *p* = 0.003). No effect on CMJ.
Romero-Arenas et al. (112)	Crossover RCT	Double-blind (Yes/Yes)	s-tDCS	17 M	22.4 ± 2.6	A: left DLPFC, C: right orbital frontal cortex	7.6*7.6	2	15	CMJ	No significant main effects or interactions for CMJ height or peak power (*p* > 0.05). ES trivial (Cohen’s d: 0.01–0.14).
Hu et al. (113)	Crossover RCT	Double-blind (Yes/Yes)	s-tDCS	12 M	24.25 ± 1.21	A: C3, C4; C: shoulders	25	2	20	CMJ	Relative peak power: η^2^ = 0.557 (*p* < 0.05), 4.9% improvement; Jump height: η^2^ = 0.088 (*p* > 0.05)
Alix-Fages et al. (114)	Crossover RCT	Double-blind (Yes/Yes)	s-tDCS/c-tDCS	25M17F	21.8 ± 2.4	A: F3; C: Fp2	7.6*7.6	2	15	30-m sprint, horizontal F-V profile	No significant differences (*p* > 0.05); ES for Pmax (a-tDCS vs. s-tDCS) = 0.20

S, surface area; I, current intensity; t, time; RCT, randomized controlled trial; M, Males; F, Females; A, anodal; C, cathode; M1, primary motor cortex; C1- C6, Cz, Fp2, The electrode positions in the International 10/20 electroencephalography system; DLPFC, dorsolateral prefrontal cortex; SJ, squat jumps; CMJ, counter movement jumps; 60-s RVJT, 60-s repeated vertical jump; SLJ, standing long jump; ES, effect size.

The potential mechanism through which tDCS may influence explosive power is thought to involve multi-level neurophysiological regulation. tDCS is believed to modulate motor cortex excitability by polarity-dependent changes in neuronal membrane potential. When a-tDCS is applied over the M1 region or supplementary motor area, it may induce depolarization of the resting membrane potential, thereby potentially lowering the threshold for action potentials generation and possibly improving the efficiency of motor commands (106, 107). This effect is evident in vertical jumps and sprints, where it improves the rapid recruitment ability of the lower limb extensor muscles. In particular, the synchronization of motor unit discharge rates is enhanced, significantly increasing the force development rate during contraction transition periods. Additionally, a-tDCS might exert long-term effects through the regulation of molecular mechanisms related to synaptic plasticity. Anodal stimulation has been suggested to enhance NMDA receptor-mediated glutamatergic transmission, promote LTP, and reduce inhibitory influences on GABAergic interneurons (11). The neurotransmitter system modulation could contribute to the strengthening of neural circuits involved in movements such as vertical jumping. Several studies also indicate that tDCS may influence cortical neural activity by modulating local cerebral blood flow, which in turn can affect motor evoked potential amplitude (108, 109). During vertical jump movements, such effects might help reduce the activation latency of spinal *α*-motor neurons, potentially improving the coordinated explosive output across lower limb joints.

However, some studies hold different views. Park et al. (110) selected volleyball players as subjects and found that a-tDCS can significantly improve the spike speed and spike consistency of professional women’s volleyball players, but it has no significant effect on vertical jump height. Rocha et al. (63) found that the vertical jump height and power of the subjects could not be improved after applying 2 mA, 15 min of a-tDCS to football players with 48 cm2. Mesquita et al. (111) applied 1.5 mA, 15 min a-tDCS to taekwondo athletes and found that the jumping ability of the subjects was not significantly improved, and their 60s fast kicking ability decreased. Romero-Arenas et al. (112) applied 2 mA, 15 min a-tDCS to the DLPFC of the subjects and found that it could not improve vertical jump height. Hu et al. (113) found that a-tDCS can significantly increase maximum output power during the vertical jump but had no effect on jump height. Alix-Fages et al. (114) also reported no significant difference in sprint ability. In high explosive power tests, vertical jump and sprint mainly depend on rapid muscle contraction and nerve coordination, which are highly limited by technical movements. Athletes typically develop a highly specialized neuromuscular control pattern compared to untrained individuals (115). When the excitability of the M1 region is enhanced by intervention, it may cause a “spillover effect” of the control signal, leading to non-specific activation of the originally inhibited antagonistic muscle motor neurons (116). Therefore, specific interventions might be needed for advanced athletes. Most studies use unilateral stimulation without significant differences; future research could explore bilateral or multi-region simultaneous stimulation to enhance effects.

Evidence suggests that a-tDCS has the potential to effectively enhance explosive power in both non-athletes and athletes, with some studies indicating that these effects can be sustained. However, for the specific enhancement of high-level athletes, particular intervention conditions might be necessary. Follow-up studies could focus on athletes in different sports to achieve better intervention results.

### 3.3 The effect of tDCS on muscle endurance

Muscle endurance refers to the body’s ability to sustain prolonged activity or to perform repeated contractions against external resistance while resisting fatigue. It plays a crucial role in both daily life and sports performance. Recent advances in science and technology have increased research interest in non-invasive neuromodulation techniques, as they could offer potential new approaches for enhancing muscle endurance.

In the study of tDCS intervention for muscle endurance, exhaustive exercise tests are commonly employed to assess performance. Time to exhaustion is a frequently used metric in these paradigms, where an increase in duration is often interpreted as a potential positive effect of tDCS on endurance capacity. Cogiamanian et al. (117) observed an increase in the time to exhaustion during a sustained 35% maximal voluntary isometric contraction of the left elbow flexor following the application of 1.5 mA a-tDCS for 10 min in 24 healthy subjects. Similarly, Williams et al. (118) reported a longer duration during a fatigue test of the elbow flexors at 20% maximal voluntary contraction after a-tDCS in 18 healthy individuals. Abdelmoula et al. (3) also found that a-tDCS prolonged the endurance time for a 35% maximal voluntary contraction of the right elbow flexor in 11 participants. In the lower limbs, Angius et al. (82) demonstrated an increase in time to exhaustion during an isometric fatigue test of the right knee extensor after 2 mA a-tDCS. However, not all observations have reached statistical significance. Denis et al. (70) used HD-tDCS on the right DLPFC of 20 healthy subjects, followed by a 30% maximum voluntary contraction isometric knee extension to exhaustion test. The results indicated no significant difference between the a-tDCS and s-tDCS groups, although the endurance duration was longer in the a-tDCS group. However, some studies have shown that tDCS may not affect the duration of continuous exercise. Kan (119), Flood (10), Radel (120), and others conducted muscle endurance tests on upper limbs, finding no significant difference in exercise duration between the a-tDCS and s-tDCS groups. Many studies, such as those by Angius (121), Barwood (122), Wrightson (123) and Isis (124), performed muscle endurance tests on lower limbs and reported no significant changes in exercise duration.

In addition, the total work and endurance index are commonly used indicators to evaluate muscle endurance levels. Increases in the total work and endurance index suggest that tDCS intervention has a positive effect on muscle endurance performance. Sales et al. (125) found that the total work during isokinetic contractions increased after a-tDCS of athletes with 2 mA for 20 min. Kamali et al. (83) applied 2 mA a-tDCS for 13 min over both the motor and temporal cortices of 12 experienced bodybuilders and reported a significant increase in the endurance index. Workman et al. (126) conducted 40 maximum torque flexion and extension tests on both knees of 31 healthy subjects. The findings indicated that the endurance index of the knee flexor muscle group increased, and the subjects tolerated 4 mA a-tDCS well. However, some studies have found that a-tDCS does not affect the total amount of work. Montenegro et al. (127) administered 2 mA for 20 min of a-tDCS on 13 subjects and observed no significant increase in total work during fatigue tests involving maximum torque flexion and extension of the knee joint.

A number of studies have suggested that tDCS may contribute to enhanced muscle endurance during exercise, and several potential mechanisms have been proposed to explain these effects. a-tDCS is thought to modulate the excitability of the motor cortex, potentially influencing the premotor area, attenuating fatigue-related muscle pain, and enhancing the descending drive to spinal motor pools, which could facilitate the recruitment of additional motor units. It has also been found that applying a-tDCS to the left temporal lobe cortex may increase the total work done during muscle contraction by regulating vagus nerve activity (i.e., parasympathetic nerve) and boosting pleasure (125). Additionally, a-tDCS has been observed to elevate blood oxygen content and cortical blood flow of the target cortex. Following stimulation, these areas often maintain elevated blood oxygen content, which correlates positively with levels of cortical excitability (128). This proposed increase in neuronal excitability and metabolic activity may reduce perceived exertion and delay task failure. To optimize the effectiveness of tDCS on muscle endurance, future research should systematically explore personalized adjustment of intervention parameters, especially the best stimulation schemes and appropriate stimulation intensities targeted at specific brain regions, to better understand its potential neural mechanisms and improve practical applications.

### 3.4 The possible mechanism of tDCS affecting muscle strength

The generation of force primarily depends on the intensity, precision, and coordination among multiple muscle groups in response to descending commands from the motor cortex. The potential for tDCS to enhance strength qualities is not considered to result from direct effects on peripheral muscle structures, but rather from the modulation of central nervous system function, which may optimize motor unit recruitment and coordination patterns (129). When a-tDCS is applied to the M1 region, the weak electric field it generates may induce depolarization in cortical neurons, potentially lowering their action potential threshold and enhancing the overall excitability of the corticospinal tract (11). This elevated baseline excitability is thought to improve the intensity and quality of descending motor commands, which could facilitate more effective recruitment of motor units innervating high-threshold fast-twitch muscle fibers. As a result, discharge frequency and synchronization among these motor units may be enhanced, thereby potentially contributing to more forceful skeletal muscle contractions within a time frame (130). The effectiveness of force production is influenced not only by muscle contractions but also by synergistic coordination among various muscles and the efficiency of force transmission through the skeletal system. Research has suggested that a-tDCS intervention may enhance muscle activation levels while reducing the co-activation levels of antagonistic muscles around the ankle joint (12). This phenomenon suggests that a-tDCS could selectively inhibit unnecessary muscle activation, potentially allowing force generated by the agonistic muscles to be transferred more effectively into external work. Furthermore, additional studies indicate that a-tDCS might improve muscle contribution rates, possibly optimizing the distribution of activation across muscles to facilitate force transmission along the kinetic chain with improved timing and proportionality (101). Wang et al. (13) reported that following a-tDCS intervention, the characteristics of the force-time curve during vertical jumps appeared to improve, alongside increased lower limb stiffness. The force-time curve was observed to transition from a unimodal to a steep bimodal profile post-intervention, reflecting that a-tDCS optimized the force application pattern and enhanced strength levels. The observed increase in lower limb stiffness suggests that the musculoskeletal system could develop a greater capacity to resist deformation and store elastic potential energy under load, with lower limb stiffness showing a positive correlation with active muscle work (131). In summary, a-tDCS may contribute to improved movement technique and coordination by modulating intermuscular synergy and force transmission efficiency, potentially leading to enhanced movement economy and muscle strength.

Some studies discussed in this review reported no significant effect of tDCS on improving muscle strength. In addition to the potentially limited impact of tDCS on specialized motor skills in elite athletes as mentioned earlier, this lack of consistent positive outcomes may be associated with individual differences in response, variations in stimulation parameters, and differences in targeted brain regions (132, 133). One proposed mechanism through which tDCS may enhance muscle strength involves the potential increase in intensity and synchronization of signals descending from the motor cortex to the downstream spinal motor neurons, as well as improving the ability of these motor neurons to recruit muscle fibers. However, neural drive is thought to have an upper physiological limit. Due to individual differences in baseline neurophysiological state, tDCS may not induce additional cortical activation once this ceiling of neural drive is approached. Consequently, increased nervous system excitability following stimulation may not necessarily translate to the recruitment of additional motor units beyond, or to a greater extent than, pre-existing capacity (86). At the same time, several studies have suggested that when the body’s muscle strength has reached a physiological plateau, the potential for tDCS to produce further enhancements may be limited (90). Professionally trained athletes typically demonstrate well-developed nerve drive capabilities and muscle strength, resulting in a relatively constrained window for additional improvement. Consequently, the effectiveness of tDCS interventions in this population appears to be more limited compared to untrained individuals. Furthermore, the effects of tDCS demonstrate notable region-specificity. Currently, the most frequently targeted areas in research are M1 and DLPFC. Studies have shown that DLPFC mainly handles higher-level cognitive functions (134), which can effectively influence subjective feelings of fatigue and effort, thus enhancing exercise duration and endurance performance. Therefore, for studies specifically investigating muscular strength and power outcomes, targeting motor-related areas such as M1 rather than DLPFC may represent a more appropriate approach, though further research is needed to establish optimal stimulation sites for different performance domains. Some of the research in this field has employed unilateral stimulation protocols, though these have not consistently yielded significant effects. Future research could therefore explore bilateral or simultaneous multi-region stimulation approaches, which potentially enhance stimulation efficacy. Given that the selection of stimulation parameters significantly impacts the outcomes, variations across studies are evident. The lower current intensity results in a reduced current density after crossing the skull to reach the target brain area. This possibly leads to only minimal changes in cortical excitability that might be insufficient to surpass necessary activation thresholds for effectively promoting motor unit recruitment.

In summary, current evidence suggests that tDCS may have the potential to enhance muscle strength in many cases. The proposed mechanisms indicate that a-tDCS appears to modulate central nervous system function by increasing the excitability of the cerebral cortex, potentially optimizing motor unit recruitment, and improving functional connectivity between relevant brain regions. In addition to the previously mentioned “ceiling” effect in athletes and proficiency in test items, stimulation parameters and individual differences appear to be significant factors contributing to the variability in tDCS outcomes. Due to the high variability in subjects’ anatomical structures (such as skull thickness), baseline neurophysiological states (such as cortical excitability levels), genetic factors, and sensitivity to stimuli (16, 17), these differences can lead to weak or no response to tDCS in some individuals, significantly reducing the overall intervention outcomes.

## 4 Limitations and suggestions

Although the review discusses existing research in this field both domestically and internationally, many studies are characterized by relatively small sample sizes, which may introduce bias and could limit the generalizability of tDCS effects across a broad population. Furthermore, significant variations in the stimulation parameters and target areas of tDCS employed in different studies complicate comprehensive analysis. Additionally, most studies have focused on the immediate outcomes, leading to a relatively limited investigation into the long-term impact of tDCS interventions.

There are significant individual differences in the stimulation effects of tDCS. The response of different individuals to tDCS may vary due to various factors, leading to uncertainty in the intervention’s effectiveness. Future research could increase the sample size and further explore the effects of tDCS, as its mechanism of action is not yet fully understood. Although some studies have proposed possible mechanisms, the deeper workings of how tDCS precisely regulates neuronal activity and produces long-term effects remain to be investigated. Future studies might utilize fNIRS technology alongside electroencephalogram and surface electromyography to simultaneously examine cerebral blood flow, EEG activity, and motor unit recruitment during and immediately after tDCS intervention. Additionally, researchers should tailor stimulation parameters to individual daily activities and neurophysiological characteristics of different populations to optimize stimulation outcomes.

## 5 Conclusion

tDCS has shown promise in modulating muscle strength based on existing research. The proposed mechanism suggests that it may induce depolarization by altering the resting membrane potential of cortical neurons, thereby potentially increasing cortical excitability, strengthening the connection between the cerebral cortex and the neuromuscular system, and facilitating more efficient muscle recruitment. Additionally, tDCS may influence an individual’s perception of effort, which could improve fatigue tolerance and extend endurance during physical performance. These potential benefits offer novel perspectives for sports training and rehabilitation; however, further research is required to refine and optimize its application. Despite the potential of tDCS to improve muscle strength, current evidence remains limited. Substantial individual variability exists in response to tDCS, influenced by differences in stimulation parameters and subject characteristics. Future research should aim to increase sample sizes, explore how individual differences affect intervention effects across different exercise groups, and identify optimal stimulation parameters to promote the application of tDCS in strength training.
